# Single-Cell Analysis of Foot-and-Mouth Disease Virus

**DOI:** 10.3389/fmicb.2020.00361

**Published:** 2020-03-05

**Authors:** Hailong Wang, Xiu Xin, Congyi Zheng, Chao Shen

**Affiliations:** ^1^State Key Laboratory of Virology, College of Life Sciences, Wuhan University, Wuhan, China; ^2^Center for Experimental Medicine, The First Affiliated Hospital of Nanchang University, Nanchang, China; ^3^Institute of Pathogenic Microorganism and College of Bioscience and Engineering, Jiangxi Agricultural University, Nanchang, China; ^4^China Center for Type Culture Collection, Wuhan University, Wuhan, China

**Keywords:** single-cell, FMDV, isolation, heterogeneity, persistent infection

## Abstract

With the rapid development of single-cell technologies, the mechanisms underlying viral infections and the interactions between hosts and viruses are starting to be explored at the single-cell level. The foot-and-mouth-disease (FMD) virus (FMDV) causes an acute and persistent infection that can result in the break-out of FMD, which can have serious effects on animal husbandry. Single-cell techniques have emerged as powerful approaches to analyze virus infection at the resolution of individual cells. In this review, the existing single-cell studies examining FMDV will be systematically summarized, and the central themes of these studies will be presented.

## Introduction

Due to technological limitations, past studies of viral infections have primarily focused on bulk cell populations. However, many biological phenomena can be overlooked when cell populations are targeted, due to the considerable cellular heterogeneity that occurs in mixed-cell populations (Cohen et al., 2008; Raj et al., 2010; Navin et al., 2011). Therefore, exploring intrinsic processes at the single-cell level is necessary. With the goal of evaluating the degree of cellular heterogeneity, single-cell research initially focused on detecting the expression of specific proteins, through simple fluorescence-activated cell sorting (FACS) analyses, but has since evolved to include complex genomic, transcriptomic, and proteomic analyses of single cells.

In recent years, single-cell technologies have been applied to the field of virology. The first study of virus-infected cell heterogeneity was performed by Delbruck, in 1940, using phage-infected *E. coli* cells. In that experiment, a significant difference in the amount of virus released from each cell progeny was observed, ranging from 20 to 1,000 phages, revealing a surprisingly broad distribution of viral yields (Delbruck, 1945). As single-cell technologies have developed, the quantification of burst sizes and the determination of infection kinetics has been performed for the vesicular stomatitis virus (VSV), which showed differences in cell-specific virus titers that span over 300-fold, suggesting a high degree of cell-to-cell variability during viral infections (Zhu et al., 2009; Timm and Yin, 2012). More recently, studies have demonstrated that, unlike the phenomena observed in multi-cellular population experiments, the RNA or virion levels of viruses, including the influenza A virus (IAV), poliovirus, and foot-and-mouth-disease (FMD) virus (FMDV), varied from cell to cell, as assessed by single-cell analyses (Schulte and Andino, 2014; Heldt et al., 2015; Xin et al., 2018).

What causes heterogeneity during viral infections? First, viral heterogeneity, including defective interfering particles (DIPs) and viral gene mutations, can alter viral infection abilities. Second, cellular heterogeneity, including cells with different cell sizes or shapes, population contexts, protein expression patterns, metabolism rates, compositions, activation status or cell cycle stages, creates specific cellular environments that can affect the success of virus progression through the cell. By combining single-cell isolation with ultra-deep sequencing, Combe et al. (2015) identified multiple, genetically diverse viral genomes within individual infectious units during a VSV infection, suggesting that an infectious unit consists of mixed virions, at least one of which has the ability to infect and replicate, while most of the others are DIPs. This observation suggested that cells are co-infected by multiple viral variants, enabling the rapid development of genetic diversity among virion progeny (Combe et al., 2015). Another study using the same virus suggested that the cell size and cell cycle of the host are factors that contribute to virus yield variability among single cells (Zhu et al., 2009), which was similar to the results reported for FMDV infections (Xin et al., 2018). However, some studies have shown that biological noise can lead to cell-to-cell variability during poliovirus and influenza A virus (IAV) infections (Schulte and Andino, 2014; Heldt et al., 2015). Biological noise concludes two parts: intrinsic noise which represents the stochastic volatility inherent to the biochemical reactions involved in the turnover of the molecules and extrinsic noise which are the fluctuations in the amount of other cellular components influencing these biochemical reactions (Heldt et al., 2015). Mathematical modeling suggests that random viral gene expression (that is, intrinsic noise) of VSV can lead to cell-to-cell variability (Hensel et al., 2009). At the same time, cellular characteristics such as the cell size and cell cycle stage (that is, extrinsic noise) were discovered to result in the titre variability observed between VSV-infected cells (Zhu et al., 2009).

To date, breakthroughs in single-cell analyses, especially single-cell omics, have been important milestones for some fields, including immunology, cancer, stem cells and virology (Cristinelli and Ciuffi, 2018; Suva and Tirosh, 2019; Tibbitt et al., 2019; Zhou et al., 2019). In the field of virology, single-cell sequencing technologies have allowed the investigation of unresolved issues such as the characterization of gene expression profiles in individual infected versus bystander cells in the mixed bulk population. Under viral infection conditions, individual cells can have specific responses to viruses that can be masked by analyses of large cell populations. The gene expression profiles resulting from high-throughput single-cell RNA sequencing (scRNA-seq) can identify changes in individual cells during viral infections, revealing vital factors that can influence the life cycle of a virus. For example, Hepatitis C virus (HCV) quasispecies were detected in individual human liver Huh7 cells by scRNA-seq (McWilliam Leitch and McLauchlan, 2013); scRNA-seq also demonstrated that the thymocyte selection-associated high mobility group box protein (TOX) was a critical regulator of CD8+ T cell persistence during chronic lymphocytic choriomeningitis virus (LCMV) infections and maintained CD8+ T cell longevity, facilitating long-term antiviral CD8+ -associated immunity (Yao et al., 2019). Through the examination of thousands of individual peripheral blood mononuclear cells (PBMCs), derived from six dengue patients and four healthy controls, multiple interferon (IFN)-response genes, particularly *MX2* in naive B cells and *CD163* in CD14+, CD16+ monocytes, were found to be upregulated in a cell-specific manner before progression to severe dengue (Zanini et al., 2018). Therefore, single-cell technology plays a significant role in the study of virology. In this review, we will evaluate the studies single-cell analysis studies that have been performed on the FMDV and discuss the results reported by these studies, to explore the mechanism underlying FMDV-associated infections.

## Single Cell Technology

### Single Cell Isolation

The single-cell technology basically includes two parts: (i) single cell isolation and (ii) single-cell analysis that can process rare biological material. The methods of cell isolation are continuedly developed from manual operations by using micropipettes or micromanipulation early to now high throughput separations such as FACS and microfluidics (Mincarelli et al., 2018).

**FIGURE 1:**
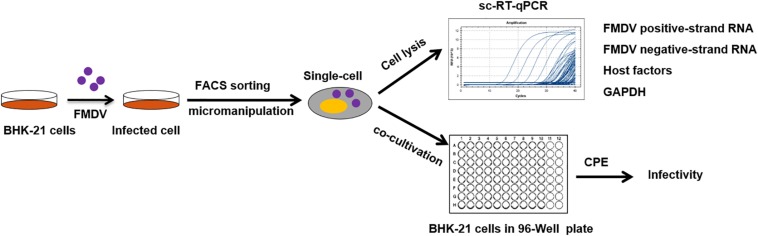
Schematic of single-cell isolation and measurement in FMDV studies. A population of adherent BHK-21 cells was infected with FMDV then trypsinized to obtain a cell suspension. The cells were sorted by FACS or micromanipulation. Intracellular RNAs of virus or host factors or GAPDH were quantified using sc-RT-qPCR. Or infected-single cells were isolated and placed in a 96-well plate (one cell per well) containing confluent normal cells. Morphologic alterations were observed under a microscope.

The earliest studies isolating single cells utilized micromanipulation in FMDV studies (Figure 1), a method for separating individual cells from cell suspensions using a micromanipulator, which is fitted with a microcapillary, attached to a microinjector, and observed through a microscope (Huang et al., 2009). Via microscopic observations, the operator selects a specific cell, moves the microcapillary in close proximity to the cell, and aspirates the cell by applying suction to the microcapillary. The volume of the aspirated liquid, including the selected cells, is then transferred to a collection container (e.g. one well of a multi-well-plate), where it is dispensed. Generally, a batch of 30 individual cells is selected within 30 min to maintain cell integrity, with minimal perturbations. Because the cells are chosen by visualization, the accuracy rate of this method approaches 100%; however, it is a low-efficiency method and requires skilled and practiced researchers.

Fluorescence-activated cell sorting is another method used to isolate single FMDV-infected cells and it represents a simple, fast, and popular technique (Figure 1; Xin et al., 2018). FACS can define different cell types among a heterogeneous population of cells, based on cell size, particle size, and fluorescence characteristics. FACS was used to analyze the impacts of host cell heterogeneity on infections by FMDV. Prior to sorting, a fluorescently labeled or stained cell suspension is prepared. The cell suspension is then pressure-driven through a flow chamber filled with a sheath fluid. The cells are aligned by the sheathing and pressure of the sheath fluid and sequentially passed through a laser beam for optical excitation. Then, optical detectors are used downstream to capture cell-specific signals. These signals depend on the physical, chemical, or fluorescence characteristics of the cells. After analysis, the cells are suspended in a closed system of small channels, and the cell stream is forced through a small nozzle with an ultra-high-frequency piezoelectric crystal. By targeted vibrational actuation after charging, the cell stream is broken into uniform small droplets, some of which carry cells. According to selected parameters, the logic circuit determines whether each droplet will be sorted, and using an electrically charged plate to deflect the droplets containing cells of interest, these droplets can be directed into a collector (generally a centrifuge tube or multi-well-plate), leaving the uncharged droplets to fall into a central waste container, achieving cell separation (Gross et al., 2015; Hu et al., 2016). The efficiency of FACS is much higher than that for micromanipulation, allowing a larger population of single cells to be sorted for different uses, with less deviation, at a single time.

Recently, microfluidic approaches for cell isolation in which cells are captured in individual droplets or nano wells for processing has been developed to meet the needs of higher throughout (Kolodziejczyk et al., 2015). Microfluidics are defined as systems based on micro-channels (10∼100 μm) and used for controlling small volume of the fluid which include several procedures (microdroplets operation, cell capture, cultivation, sensing, sorting, lysis and omics analysis) for single-cell operation (Zhu et al., 2018). On account of the ability of controlling and manipulating fluids in the range of micro to pico-liters, microfluidics has been as a platform-level and continuously evolving technology for single-cell processing and analysis (Dusny and Grunberger, 2019). In single cell studies, microfluidics have many incomparable advantages over conventional techniques. First, microfluidics has the less reaction volume so that it could improves the sample concentration, which facilitates the reaction and reduces the analysis time. Second, microfluidics permits that a series of manipulations are directly conducted in sequence on a single chip, avoiding the waste of sample in the process of transferring between tubes. Further, well-designed chambers and controllable operations of microfluidics offer the favorable conditions for high-throughput single-omics analysis (Luo et al., 2019). Besides, laser-capture microdissection (LCM) are sometimes applied to acquire the rare single cells directly from tissue slices but it is low throughput and time-consuming (Aaltonen et al., 2011).

### Single Cell Omics

After isolating single cell, how to measure internal biomaterials such DNA, RNA and proteins to get hidden information of individual cell is another tremendous challenge. The great development of next-generation sequencing technologies which now are capable to detect genome, epigenome, transcriptome, or protein profiling of single cell has revealed heterogeneity-related molecular driving forces for cell subtype classification and physiology identification (Macaulay et al., 2017). Therefore, single-cell genomics, epigenomics, transcriptomics, proteomics and multiomics come into being as a result.

It is well-known that the genome is relatively stable throughout life and remains the same independently of cell type. However, over time, during cell division, exposure to external factors such as radiation or chemicals can cause random mutations within the genome and bring about the development of disease (De, 2011). Single-cell genome analysis illustrates the heterogeneity in inheritance and aberrance information at single-cell level. Because there is just approximately 7 pg of genomic DNA in a diploid human cell which is not enough to sequence, several approaches such as multiple displacement amplification (MDA) multiple annealing and looping-based amplification cycles (MALBAC) and linear amplification via transposon insertion (LIANTI) to amplify single-cell DNA have been applied (Chappell et al., 2018). However, in this process some artificial errors also are introduced such as locus and allelic dropouts, as well as unevenness in amplification, generation of chimeric DNA molecules, and introduction of base copy errors (Gawad et al., 2016). For reducing the negative effects caused by the errors, new methods for single-cell genome sequencing are emerging. For example, Linear amplification via transposon insertion (LIANTI) which utilized transposases loaded with a T7 promoter containing adaptor reduces amplification bias and errors associated with non-specific priming and exponential amplification in conventional methods (Chen et al., 2017); single-stranded sequencing using microfluidic reactors (SISSOR) which is a microfluidics device to separate the Watson and Crick strands of chromosomes present in the lysate of a single cell enabled sequencing of single-cell genomes with error rates as low as 10^–8^ (Chu et al., 2017). These single-cell genomics technologies have been used to diverse fields of biological research: (a) detecting acquired drug resistance mutations; (b) discovering clonal evolution, tumor heterogeneity, and cell of origin; (c) characterize stem cells in solid tumors and circulating tumor cells (CTCs), and (d) unscrambling complex biological processes such as those of the central nervous and immune systems (Lee, 2019).

Sometimes, the cell types are not determined by genome but the modifications of the genome such as DNA methylation, histone modification, chromatin accessibility, and chromosome conformation. Epigenomics analysis which detects how genomic structure changes influences cellular phenotype is increasingly recognized as markers of embryo development, disease and cancer (Rivera and Ren, 2013). DNA methylation, including cytosine methylation (5mC), hydroxy methylation (5hmC) and formyl cytosine (5fC) which can regulate gene expression are measured through single-cell bisulfite sequencing (scBS-seq), single-cell 5hmC sequencing (scAba-seq) which uses the restriction endonuclease AbaSI to induce double-strand breaks in modified DNA sequences, followed by adaptor ligation, amplification, and library preparation and single-cell 5fC sequencing (chemical-labeling enabled C-to-T conversion sequencing, CLEVER-seq) (Wang and Navin, 2015). Modifications to histone proteins can be surveyed at single-cell resolution using droplet-based chromatin immunoprecipitation (Drop-ChIP) which identifies histone H3 demethylation at lysine 4 (H3K4me2) and histone H3 trimethylation at lysine 4 (H3K4me3) (Rotem et al., 2015). In addition, Single-cell high-throughput chromosome conformation capture (scHi-C) can be used to determine the chromosomal architecture within single cells, revealing thousands of contacts and to associate dynamics in topologically associated domains with cell cycle progression (Ramani et al., 2017).

Single-cell transcriptome analysis reflects the gene expressions across specific time and space, which helps to determine the subpopulation, discover novel cell types and reveal the varying cell states. Currently the protocols of single cell RNA sequence (scRNA-seq) all follow workflow: single cells isolation; single cells lysis; reverse transcription into cDNA; pre-amplification; libraries preparation for sequencing and downstream analysis (Wu et al., 2017). Scale is a key variable in scRNA-seq experiments with the development of isolation methods from medium throughput on FACS-sorted cells isolated into multi-well plates to high throughput on microfluidics-based approaches which enabled thousands of cells to be processed in parallel. The methods of scRNA-seq conclude: Smart-seq/Smart-seq2, which could be able to amplify full-length of single-cell cDNA not only compute transcript abundance within the cell, but also explore sequence variation within the transcriptome (Picelli et al., 2013); CEL-seq/CEL-seq2, which relies on IVT-based amplification and 3’ enrichment, generates 3’-end cDNA libraries, incorporating unique molecular identifiers (UMIs) – random barcodes added to the sequence during the first reverse transcription (Hashimshony et al., 2016); STRT (single-cell tagged reverse transcription), which is Template-switching PCR-based full-length transcript amplification followed by 5’selection (Islam et al., 2011); Droplet-based approaches such as Drop-seq, InDrops, 10X Genomics, Chromium and Dolomite Nadia, which cells are partitioned into individual droplets and cDNA molecules are uniquely barcoded during reverse transcription (Macosko et al., 2015); Nanowell approaches such as SeqWell, Nanogrid (ICell8) and BD Rhapsody, which cells are partitioned into in dividual wells of a custom built nanowell chip and cDNA molecules are uniquely barcoded during reverse transcription (Gierahn et al., 2017). The latter two methods facilitate large-scale cell numbers at low cost and have been commercialized.

Proteins are key executors of biological processes and connect genomic information to biological functions. But the advance of single cell proteomics is lagged far behind single cell genomics and transcriptomics because there exit huge challenges such as the enormous proteome complexity, the lack of suitable antibodies and low concentration of proteins in single cell. At moment, flow cytometry is widespread method for single-cell protein analysis based on fluorophore-labeled antibodies and is capable of analyzing up to 50 parameters in parallel. When antibodies are labeled with heavy metal ion tags instead fluorophores which are read out using time of-flight mass spectrometry, the method called CyTOF allow more than 40 proteins to be multiplexed (Spitzer and Nolan, 2016). An alternative method, antibody barcoding with photocleavable DNA (ABCD) technique which uses DNA barcode-tagged antibodies and protein levels can be determined by quantitative PCR or sequencing of the conjugated DNA can increases in multiplexing (Ullal et al., 2014). Compared with antibody-dependent technologies, single-cell proteomics by mass spectrometry (SCoPE-MS) increases the depth of single-cell proteomics with 583 proteins quantified at the single cell levels (Budnik et al., 2018). Based on Microfluidics technologies, microchip-based proteomics analysis is able to simultaneously quantified up to 40 nuclear, cytoplasmic, membrane and secreted proteins across thousands of single cells, with the sensitivity threshold of as low as a few hundred protein copies per cell (Yang et al., 2016).

The property of an individual cell is not determined by any isolated factor but decided by the complex interplay of molecules with in its genome, epigenome, transcriptome and proteome. Therefore, the emergence of single cell “multi-omics” approaches which aim to simultaneously assay different types of molecules, such as DNA and RNA or RNA and protein is meaningful (Yang et al., 2016). At present, there are two methods that permit both genome and transcriptome sequencing from single cells: DNA–RNA sequencing (DR-seq) which preamplifies gDNA and mRNA simultaneously before dividing the reaction in two for constructing the gDNA and mRNA libraries separately, and another method is genome and transcriptome sequencing (G&T-seq) that mRNA is physically separated from gDNA using oligo-dT-coated beads to capture and isolate the polyadenylated mRNA molecules from a fully lysed single cell (Dey et al., 2015; Macaulay et al., 2016). At same time, new methods such as single-cell triple-omics sequencing (scTrio-seq) and Single-cell methylome and transcriptome sequencing (scMT-seq) that linking epigenomic and transcriptomic measurements of the same single cell allows exploration of the regulatory mechanisms are established (Hou et al., 2016). The G&T-seq method has further been adapted to incorporate NOME-seq analysis of the same single cell, which giving a triple readout of chromatin accessibility, DNA methylation, and gene expression (Clark et al., 2018). Two methods, named cellular indexing of transcriptomes and epitopes by sequencing (CITE-seq) and RNA expression and protein sequencing assay (REAP-seq), similarly combine the Drop-seq/Indrops platform with DNA barcoded proteins for simultaneous analysis of single-cell proteins and transcriptome which is useful to reveal the information of genetic expression and actual phenotype (Peterson et al., 2017; Stoeckius et al., 2017).

Except above mentioned methods, single cell spatial transcriptomics and metabolomics are developed in recent years. Single spatial transcriptomics which measure the surroundings of single cell, especially within a primary tissue or organoid model, is necessary to understand the correlation of a single cell’s phenotype with its spatial information. For example, sequential fluorescence *in situ* hybridization (seqFISH) approaches could accurately detect transcripts from thousands of genes, which are connected with their cellular locations (Shah et al., 2016). Metabolome defined as the full collection of all low molecular-weight metabolites that are produced by a cell, could be a key indicator of cell state – reflecting the precise metabolic activity and condition within the cell. Measurement techniques including mass spectrometry, capillary electrophoresis, optical spectroscopy and fluorescence detection have used in single-cell metabolomics (Zenobi, 2013). It was reported that analytical validation of a single-cell metabolite analysis using the microarrays for mass spectrometry (MAMS) platform has also been applied to monitor cellular responses upon environmental and genetic perturbation (Ibanez et al., 2013). Given the significance of the single cell omics, it is it is anticipated more advanced and mature technologies appear with higher throughput and few costs.

## FMD and FMDV

Foot-and-mouth-disease is an acute, highly contagious disease of cloven-hoofed animals and is listed as the first Class A infectious disease by the World Organization for Animal Health (OIE) (Rodriguez and Gay, 2011; Verma et al., 2012; Ding et al., 2013; Xu et al., 2013). FMD can affect cattle, buffalo, pigs, sheep, goats, and approximately 70 species of wild animals (Verma et al., 2008; Teifke et al., 2012; Verma et al., 2012). This disease can be found almost everywhere in the world where animals are raised and causes huge economic losses (Ferrer-Orta et al., 2009; Chakraborty, 2014; Al-Hosary et al., 2019; Kim et al., 2019). For example, in 1997, an FMD epidemic resulted in more than 4 million pigs being killed in Taiwan (Kitching, 1998). The disease presents with sores that emerge on the mouth, tongue, nose, nipple, toes, and other hairless areas of the skin. The disease develops within 2–3 days post-exposure, and the disease course lasts from 7–10 days (Burrows et al., 1981; Zinna, 2002; Teifke et al., 2012).

The etiological agent of this disease, FMDV, is a member of the genus Aphthovirus in the family Picornaviridae (Belsham, 1993). Thus far, seven distinct serotypes (A, O, C, Asia1, and South African Territories 1, 2, and 3) have been identified, with a wide range of subtypes existing within each serotype (Knowles and Samuel, 2003). FMDV is approximately 30 nm in size and belongs to the single-stranded positive-sense RNA virus family, with no envelope and icosahedral symmetry. The viral genome is approximately 8.5 Kb, encoding the structural proteins viral protein (VP)1, VP2, VP3, and VP4 and at least 10 non-structural proteins, including L, 2A, 2B, 2C, 3A, 3B1, 3B2, 3B3, 3C, and 3D and some intermediate precursors (Jamal and Belsham, 2013). The VP1-4 proteins form virions, and the most immunogenic protein, VP1, has maximal exposure on the capsid surface, which is crucial for FMDV absorption and entry into cells (Thomas et al., 1988; Lea et al., 1994). The contribution of VP3 is primarily to stabilize the capsid. The polyproteins 3A, B, and C and 3D are non-structural proteins that play important roles in viral replication, and the 3D protein is an RNA-dependent RNA polymerase (Ferrer-Orta et al., 2009; Gu et al., 2009).

Foot-and-mouth-disease virus can cause both acute infections and asymptomatic, persistent infections that follows the acute phase of the infection (Martin-Acebes et al., 2010; Kopliku et al., 2015; Stenfeldt et al., 2016). In the latter case, animals become carriers of the virus, and the persistence of the virus varies from species to species. FMDV lasts for 12 months in cattle, 6–9 months in sheep, and 4 months in goats, while African buffalo can remain carriers for as long as 24 years (Chakraborty, 2014). A low level of FMDV excretion from the pharynx of ruminants was found in these carriers, which is also associated with the species and virus lineage. Under natural conditions, FMDV from African buffaloes is able to transfer to cattle and impala, and the injection of saliva obtained from carrier animals into cattle and pigs has caused infections under experimental conditions (Klein, 2009; Bao et al., 2011). Therefore, understanding how FMDV hides in the host during persistent infections and determining the differences between uninfected cells and infected cells is necessary but difficult to determine at the population level. The emergence of single-cell technologies provides the opportunity to discover relationships between various cells and FMDV.

## Single-Cell Detection Methods for FMDV

Individual cells are reported to weigh 3–4 ng, to occupy a volume of approximately 1 pL, and to have diameters of approximately 10 μm, depending on the cell type. The total protein content of a single cell is approximately 700 pg, but the concentration can vary by up to seven orders of magnitude (Beck et al., 2011). The quantity of genomic DNA in a single cell is estimated to be approximately 6 pg, while the quantity of RNA is approximately 10 pg (Han and Lillard, 2000; Schmid et al., 2010). The detection of FMDV mRNA, protein and virions can be difficult after single-cell separation because these components represent very small proportions of the total cell protein, DNA, and RNA populations. In 2008, a novel single-cell quantitative real-time reverse transcriptase polymerase chain reaction (sc-qRT-PCR) method, with high sensitivity for the detection of viral RNA copies greater than 10 per cell, was developed to analyze FMDV RNA levels (Figure 1). To ensure isolation and detection of single cells, glyceraldehyde 3-phosphate dehydrogenase (GAPDH) is used as a positive control that can be measured in almost every single cell. During the acute infection assay, a total of 224 single cells were isolated, lysed, and quantified. Among them, 185 samples were positive (i.e. the FMDV RNA copy number was greater than 10), and the number of viral RNA copies ranged from 10 to 1,000,000. The ratio between viral-RNA-positive and -negative cells was 82.6%. The FMDV genomic RNA copy number in most positive samples ranged from 1,000 to 10,000 (Huang et al., 2009). This was the first report to use sc-qRT-PCR for the detection and quantitation of viral genomic RNA, rather than host mRNA. This method enables the study of viral replication and its relationship with host cells at the single-cell level.

To detect FMDV negative-strand RNA, which is replicative, intermediate, and important to viral replication, a simplified, cost-effective, two-step duplex qRT-PCR assay was developed to detect, and quantify FMDV positive-stranded RNAs and negative-stranded RNAs, simultaneously, in individual cells, which can reduce the time, labor, and sample quantities necessary and is especially useful for small samples, such as tissue samples and single-cell samples (Li et al., 2009). The two primer-probe sets used in the duplex qRT-PCR were designed within the coding regions of the 2B gene and the 3D gene, which have the fewest variations among serotypes, allowing this assay to be used universally for the quantitation of virus strains belonging to the FMDV O, A, C, and Asia 1 serotypes. The authors tested 187 FMDV-infected single-cell samples, of which 55 cells were positive based on both positive- and negative-strand RNA qPCR, and the ratio of positive- to negative-strand RNA ranged from 15.6 to 1,463.4. The differences among single-cell samples were significant, indicating that active viral replication differs greatly among individual cells.

Infectivity is another important target of virus detection. A co-cultivation method was used, in which single cells were isolated and directly placed into individual wells of a 96-well plate (one cell per well) containing confluent normal cells to demonstrate the infectivity of the viral loads in single cells, which were sorted from an FMDV-infected cell population. The formation time and intensity of the cytopathogenic effect (CPE) can represent the infective ability of a virus (Figure 1; Huang et al., 2009; Xin et al., 2018). These results also demonstrated cell heterogeneity for FMDV infectivity.

## Discovery in FMDV Research With Application of Single-Cell Technology

At present, the single-cell analyses of viruses primarily focus on two aspects: cell-to-cell variability during the replicative cycle of a virus and different host cell responses to viral infections. Through the isolation and detection of single cells from cell populations, the expression levels of many viruses, such as VSV, IAV, HCV, and Hepatitis B virus (HBV), have been found to vary across individual cells. To better understand this phenomenon, single-cell omics have been used to reveal how the mRNA and protein profiles of host cells change under viral invasion conditions. The innate immune response is the first line of defense against viral infections. For example, West Nile virus (WNV)-inclusive single-cell RNA sequencing has demonstrated considerable transcriptional heterogeneity in the IFN-I response to viruses, and IFN-stimulated genes were negatively correlated with viral RNA abundance (O’Neal et al., 2019). Another single-cell study has shown that the induction of type I IFN by herpes simplex virus (HSV-1) is restricted to a rare sub-population of abortively infected cells, which is in contrast to other studies examining the IFN-I response to viral infections (Drayman et al., 2019). In the field of FMDV, single-cell analyses have also focused on cell heterogeneity during FMDV infections, especially persistent infections.

In 2011, an *in vitro* model of persistent infection, using the FMDV serotype O (FMDV O) in BHK-21 cells, was established. In the study, BHK-21 cells infected with FMDV O were maintained in medium containing ammonium chloride. Surviving cells were isolated using a micromanipulator to form single-cell clones, and 17 positive-cloned cell strains were obtained, which each represented a model of persistent infection (Huang et al., 2011). In these cells, FMDV RNA, proteins, and viral particles were detected. Unexpectedly, unlike the wild-type virus, the virus released from these cells had lost the ability to form plaques but could still infect host cells. The establishment of a persistent cell culture can provide a system for elucidating the mechanisms underlying viral persistence. On the basis of this system, a relationship between FMDV infections and the host emopamil-binding protein (EBP) gene was identified, through the global transcriptional analysis of single cells isolated from a model of persistent FMDV infection (Fang et al., 2017). In the study, 231 individual persistently infected BHK-21 cells were isolated by micromanipulation after passages 28, 38, and 68 (PI28, PI38, and PI68, representing early, middle, and late infection, respectively), and were used to investigate variations in the expression levels of the FMDV virus 3D gene and the host EBP gene by sc-qRT-PCR. The results showed that the proportion of single cells carrying viral RNA was the lowest and the mean EBP expression level was the highest in PI28 cells, In PI38 cells, EBP gene expression gradually decreased, the proportion of single cells carrying viral RNA increased (98.7% of the total), and the expression level of the viral 3D gene in single cells reached the peak among all generations. In PI68 cells, the proportion of single cells carrying viral RNA was 95.8%, the expression level of FMDV 3D gene decreased to the lowest level, and the expression level of host EBP gene declined rapidly, reaching a minimum. These results in single cells showed that the establishment of a persistent FMDV infection in cells represents a dynamic process, in which the EBP gene plays a vital role, and is the result of mutual adaption between virus and host cells.

The model of persistent FMDV infection represents a mixed population, with individual cells containing various levels of FMDV RNA and some cells completely free of viral RNA. To investigate the mechanism of virus-cell co-evolution, a traditional single-cell isolating method, the limiting dilution-based method, was applied to obtain special cell components (Han et al., 2018). Persistently infected cells were serially diluted in 96-well plates, with a cell number between 1 and 10 for minimal inoculation. After culturing for 1–2 weeks, wells with monoclonal cells and supernatants from FMDV-negative cells were screened and further expanded in culture. The evolved FMDV-negative cells, which were named “BHK-VECs,” showed resistance to the parent FMDV, due to the complete blockage of viral RNA replication, making the virus proliferation process ineffective and suggesting that the host cells had evolved. However, a persistent infection could be established in these cells when infected with FMDV from the model of persistent infection, which is named FMDV-Op, suggesting that FMDV-Op had adapted to the evolutionary environment. In addition, the virus displayed a lytic infection in BHK-21 cells, suggesting that the establishment of a persistent FMDV infection was not due to the generation of replication-defective and non-cell-lysing viral mutants, but rather to select host cells that were resistant to viral infection, to limit the cell disease. The BHK-VECs are first reported in BHK-21-derived cell lines that have the ability to resist FMDV infections and are generated from the environment of virus-host co-evolution. These findings are directly attributable to single-cell technology and would likely be masked during analyses of cell populations.

In a cell population, FMDV RNA levels fluctuate in different cells because of cell heterogeneity (Huang et al., 2009; Li et al., 2009; Fang et al., 2017; Xin et al., 2018). Cells have many characteristics, but which of these influences FMDV replication? In a recent study, cell size, cell inclusion, and cell cycle were suggested to affect FMDV infection, based on single-cell analyses (Xin et al., 2018). Using FACS, FMDV-infected BHK-21 cells, at a multiplicity of infection (MOI) of 0.0001 or 3, were sorted into single cells, either 24 h post-infection (hpi) or 6 hpi, and then the RNA levels and FMDV infectivity levels of each cell were analyzed by sc-qRT-PCR and co-cultivation. The results demonstrated that cell size (represented by forward scatter [FSC]) and cell inclusion number (represented by side scatter [SSC]) were positively correlated with RNA levels and the infective abilities of FMDV. Interestingly, when the bulk cell population was divided into three parts, based on cell size or cell inclusion number after FMDV infection, similar results were found. Larger cells or cells with more inclusions may contain additional resources for conducting biological reactions that are beneficial to viral infection and replication. Furthermore, the levels of most proteins and mRNAs and the sizes or numbers of organelles reportedly increase with increased cell size, and viruses require these resources to replicate. In addition, cells in the G2/M phase contained more viral RNAs and a higher percentage of 3D-positive cells than cells in other phases. Furthermore, using drugs to arrest the cell cycle in different phases revealed that cells arrested in the G2/M phase had increased replication efficiency and favored the production of viral progeny. A similar result was observed during persistent infection, with larger cells, cells with more inclusions, and cells in the G2/M phase containing more FMDV RNA.

A viral adsorption experiment showed that the absorbed FMDV RNA contents in large cells and cells with high numbers of inclusions were greater than those in cells that were smaller and had fewer inclusions, suggesting that cell size and inclusion numbers affected the FMDV adsorption processes. FMDV receptors, especially αvβ6, which are crucial to adsorption, were closely associated with cell size, the number of inclusions, and cell cycle. Surprisingly, through FACS analysis, cell size, the number of inclusions, and cell cycle also have intrinsic connections with each other, implying that FMDV prefers to infect specific populations of host cells. The results of this study also suggested that host cell heterogeneity influenced the adsorption of FMDV due to differences in the levels of FMDV integrin receptor expression (Xin et al., 2018).

## Conclusion

The advent of single-cell technology has revealed the various fates of individual cells under the conditions of viral infection. However, what drives the complicated response programs in individual cells requires further exploration. For FMDV, cell-to-cell variety has been shown to exist during both acute and persistent FMDV infections, based on single-cell analyses. In addition, cell size, cell inclusion numbers, and cell cycle may determine the susceptibility of host cells to infection by FMDV (Xin et al., 2018). However, these findings represent the tip of the iceberg, and deeper mechanisms must be uncovered. For example, whether the response to FMDV infection is different in each cell and what key factors determine these responses remain unclear. What makes FMDV capable of latently infecting particular cells and what makes them break out? Single-cell genomics, transcriptomics, and proteomics may resolve some of these issues, however, relevant studies of FMDV are currently lacking. Single-cell sequencing may help us characterize cellular heterogeneity and identify the factors that influence FMDV infections, especially persistent infections. For example, we plan to isolate single cells from established persistent FMDV cell lines which are mixed population with virus-infected cells and virus-uninfected cells and find the difference of individual gene expression profiles through single-cell RNA sequence which could be useful to reveal the important factors or signaling pathway influencing FMDV infection. In addition, a small part of cells may be evolved in the process of defending against virus and using the single cell isolation combined with genomics and epigenomics analysis could seek for these cells in the virus-infected cell populations and identify their characteristics which may be helpful to invent antiviral strategies. Besides, we could use single cell technology to separate large of single cells from the different tissues of FMDV-infected animals, and then find susceptible cell populations with different degrees and search the internal correlation of expression profiles of cells with virus through single omics analysis. Virus could not only change the expression but also the location of some host proteins. Using single spatial transcriptomics to detect these proteins under FMDV-infected or FMDV-uninfected conditions would be useful to know which proteins are recruited to which special place in different replication stages that is important to realize the mechanisms of virus infection. Therefore, establishing a system for the single-cell sequencing of FMDV-infected cells and obtaining expression profiles using single-cell analyses represent important goals for FMDV research. In the future, as single-cell analyses in FMDV-infected populations increase, the mechanisms underlying FMDV infection will gradually be unmasked.

## Author Contributions

HW and XX wrote the draft manuscript. CS and CZ gave instruction and proof-read the manuscript.

## Conflict of Interest

The authors declare that the research was conducted in the absence of any commercial or financial relationships that could be construed as a potential conflict of interest.
